# Out of the classroom and into the community: medical students consolidate learning about health literacy through collaboration with Head Start

**DOI:** 10.1186/s12909-016-0635-z

**Published:** 2016-04-23

**Authors:** Emily Milford, Kristin Morrison, Carol Teutsch, Bergen B. Nelson, Ariella Herman, Mernell King, Nathan Beucke

**Affiliations:** University of Missouri School of Medicine One Hospital Drive, MA204, Columbia, MO 65201 USA; University of California Los Angeles Anderson School of Management, 110 Westwood Plaza, Los Angeles, CA 90024 USA; Mattel Children’s Hospital and Department of Pediatrics, David Geffen School of Medicine at UCLA, 10833 Le Conte Ave, Los Angeles, CA 90095 USA; UCLA Children’s Discovery and Innovation Institute, 10833 Le Conte Ave., Los Angeles, CA 90095 USA; Central Missouri Community Action, 807 B North Providence Road, Columbia, MO 65203 USA; University of Missouri School of Medicine Department of Child Health, 400 N. Keene Street, Suite 010, Columbia, MO 65201 USA

**Keywords:** Health literacy, Medical student education, Service learning, Pediatric obesity prevention

## Abstract

**Background:**

Medical schools need to teach future physicians about health literacy and patient-doctor communication, especially when working with vulnerable communities, but many fall short. In this article, we present a community-based, service learning experience over one academic year during the pre-clerkship portion of medical school as an innovative and successful model for medical students to learn about health literacy and practice effective communication strategies. “Eat Healthy, Stay Active!” (EHSA) is a 5-month pediatric obesity intervention designed for Head Start children, their parent (s), and staff. We hypothesized students’ attitudes, knowledge, and skills confidence regarding healthy literacy and patient communication would improve from baseline after receiving training and serving as family mentors in the EHSA intervention.

**Methods:**

First- and second-year medical students were trained through a series of didactics and then partnered with Head Start children, parents, and staff to help educate and set goals with families during the EHSA intervention. Medical students were given a pre- and post-intervention survey designed to measure their attitudes, knowledge, and skills confidence regarding health literacy. The pre-survey was administered before the first didactic session and the post-survey was administered after the conclusion of the EHSA intervention. We compared students’ pre- and post-intervention responses using paired t-tests. Throughout the project, the medical students were asked to complete a set of open-ended journal questions about their experiences. These responses were examined using qualitative, thematic analyses. Additionally, the Head Start parents and staff were asked to complete a survey about their experience working with the medical students.

**Results:**

Participant (*n*=12) pre- and post-surveys revealed that medical students’ attitudes about the importance of health literacy were ranked highly both pre- and post- intervention. However, knowledge and skills confidence regarding health literacy showed statistically significant improvement from baseline. Journal entries were categorized qualitatively to demonstrate medical students’ insight about their growth and development throughout the project. Survey results from Head Start parents showed medical student participation to be highly valued.

**Conclusion:**

Providing medical students with a service learning opportunity to work with individuals with low health literacy in their pre-clerkship years increased students’ knowledge and skills confidence regarding health literacy and communication.

## Background

### Teaching health literacy in medical schools

Health literacy, defined as the degree to which individuals have the capacity to obtain, process, and understand basic health information and services needed to make appropriate health decisions [1], is a crucial element in training future physicians because poor health literacy has been shown to be a determinant of poor health outcomes and higher costs [2–6]. In 2010, the Department of Health and Human Services Office of Disease Prevention developed the National Action Plan to Improve Health Literacy, which includes seven goals and suggests strategies for implementation, including a focus on education for health professionals [2, 4]. The Institute of Medicine also recommends teaching medical students about health literacy, but currently standards are not clearly established for implementing health literacy training into medical school curricula [3, 6, 7]. This has resulted in very little known about how to effectively teach health literacy issues to future physicians [7]. Coleman and colleagues published a consensus study about health literacy practices and educational competencies for health professionals, which included recommendations about attitudes, knowledge, and skills that ought to be taught to health professionals [8]. A common strategy involves including up to three hours of didactic sessions about health literacy and patient-doctor communication during the first two (pre-clerkship) years, as well as simulation experiences, standardized patients, and role play [3, 6, 7, 9]. A project done at Harvard Medical School suggests the novel strategy of partnering medical students with community health literacy learners as an effective way for medical students to gain a better understanding of health literacy issues while serving the community [5]. This paper discusses the significant innovation of using an academic-yearlong service learning experience to teach health literacy to medical students.

### Collaboration with the “Eat healthy, stay active!” (EHSA) intervention for Head Start

Head Start is a longstanding program of the United States Department of Health and Human Services that provides comprehensive early childhood education and services for low-income children and their families. Nationally during 2011–2012, there were 1.14 million children enrolled in Head Start programs. Head Start enrollment guidelines require families to be living in poverty, with at least 90 % of families living at or below 100 % of the federal poverty level ($23,550 annually for a family of four in 2013). Demographics of Head Start families overlap with those that predict low health literacy [2].

Developed at the UCLA/Johnson & Johnson Health Care Institute at the Anderson School of Management (HCI), EHSA is an obesity prevention intervention that utilizes a tri-level approach involving Head Start children, parents, and staff. Its goal is to implement an engaging low literacy curriculum around healthy eating behaviors and increased physical activity levels coordinated across the three cohorts. Data from its pilot intervention, involving six Head Start grantees across eight states, demonstrated statistically significant reductions in BMI among the staff, children, and parents who participated, with children’s weight changes correlating with their parents’ weight changes [10]. The intervention involves educating staff and parents about nutrition and active lifestyles during interactive sessions as well as incorporating healthy food choices and activities into Head Start classroom curricula for children. There are additional workshops and outings held after the core training to reinforce the learning. Participants’ anthropometric measurements and survey responses about knowledge, skills, health literacy, and behaviors were analyzed pre- and post-intervention. Due to the high obesity rates in their population, Central Missouri Community Action (CMCA) Head Start partnered with HCI and University of Missouri School of Medicine (MU SOM) to pilot the EHSA intervention in their community for the first time. Incorporating medical students into the EHSA intervention has never been done in the past.

The aim of our investigation was to measure if an academic-yearlong, intensive, community-based, service learning experience during pre-clerkship medical school years could improve students’ attitudes, knowledge, and skills confidence in health literacy. We hypothesized students’ attitudes, knowledge, and skills confidence regarding healthy literacy and patient communication would improve from baseline after receiving training and serving as family mentors in the EHSA intervention designed for low-health literacy learners. In a mutually beneficial arrangement, we hoped to provide the Head Start community with young, motivated volunteers working to improve health literacy of the community and foster families’ confidence is talking to health care providers.

## Methods

### Creating a partnership

Coordinating the EHSA intervention and medical student involvement was a voluntary effort and required significant motivation from all involved parties. A guiding coalition was formed to assure broad community participation, input, and funding. The membership of the guiding coalition included representatives from CMCA, MU SOM, HCI at UCLA (developers of EHSA), Health Literacy Missouri, Boone County Health Department, and other community health care professionals (pediatricians, dietitians, nurses) who met quarterly to help design the intervention. This coalition provided a working model on how to engage the larger community in one’s efforts. The diverse perspectives from all coalition members (including medical students) helped create a role for medical students in the project that would maximize learning opportunities and use of health literacy skills. CMCA created a committee of consisting of a nurse, a nutritionist, an early childhood education specialist, and classroom teachers who have worked extensively with the Head Start population in Mid-Missouri in order to tailor the EHSA intervention to meet the specific needs of the community. Additionally, the minutes of each coalition meeting were discussed with the Head Start Policy Council (governing body of Head Start parent representatives) and their input was sought in curriculum design and implementation strategies.

### “Eat healthy, stay active!” as a service-learning project

Developing a service-learning project that would be attractive to medical students was crucial to the success of this collaboration. Participation was designed as an extracurricular activity at MU SOM and did not interfere with existing medical curriculum. The medical students and the pediatrician faculty advisor provided volunteer hours that made the EHSA possible with the limited resources available to CMCA Head Start. At implementation of the project, no other available opportunities at MU SOM provided a long-term structured, volunteer experience with children and families in spite of expressed students’ desire for such an experience. Medical students were involved with the project design because they have a first-hand understanding their schedule’s demands and MU SOM’s culture and mission. First- and second-year medical students at MU SOM were eligible to participate in the project. Interested students were invited to submit an application consisting of a set of short-answer questions. Applications were evaluated on enthusiasm to learn, prior experience, and openness to cultural diversity.

The selected medical students attended weekly didactic sessions during evening hours over the two months prior to initiation of the EHSA intervention with families. The topics covered were: (1) Head Start 101 (background about the mission of Head Start and the population it serves), (2) the Culture of Poverty (statistics about poverty in the US and poverty-created barriers to healthcare access), (3) Nutrition for Children (presented by a registered dietician), and (4) Health Literacy Skills Training, including how to administer the Newest Vital Sign (NVS), a validated health literacy measurement tool [11–13]. After the completion of didactics, the medical students served as educators and mentors at staff and parent trainings and in the Head Start classroom. Defined roles of the medical students were to (1) visit their assigned Head Start site and measure their assigned children’s pre-intervention BMI, (2) participate in an EHSA activity with children in the Head Start classroom, (3) attend core parent trainings, where they (4) administered the Newest Vital Sign (NVS) to assess baseline parent and staff health literacy, and (5) measured and explained BMI findings to their assigned staff and parents (child BMI was also given to the parents). Students explained BMI numbers, avoiding medical jargon, using carefully chosen language such as “healthy weight” and “unhealthy weight”. The students then (6) led one-on-one nutrition and exercise goal setting sessions with parents. The students also participated in the training and activities alongside the staff and parents to answer questions and continue to motivate. One student who was proficient in Spanish worked alongside the CMCA translator with the Spanish speaking families. Students were encouraged to engage with their assigned families through the EHSA Facebook page with health-literate nutrition and exercise tips, recipes, and reminders about EHSA activities and contests. At the completion of the 5-month EHSA intervention, the students helped to host a graduation celebration for children, parents, and staff where they collected all post-measurements and post-NVS surveys. Over 7 months (approximately an academic school year), the students volunteered an average of 2 h per week, excluding weeks of examinations or holidays. The pediatrician faculty advisor attended all activities to serve as a mentor and resource for unexpected situations for the students. During these interactions, the medical students were given an opportunity to use health literacy skills learned in didactics (such as plain language and teach-back) and instructed to take time to understand the family situation, identify barriers, provide education, and use motivational interviewing techniques to help the families with their healthy lifestyle goals.

### Surveys

The cohort of participating medical students’ attitudes, knowledge, and skills confidence regarding health literacy were compared pre- and post-intervention with surveys. Surveys consisted of a combination Likert Scale and multiple-choice questions. The pre-survey was administered after acceptance into the project before the didactic sessions. The post-survey was given approximately 7 months after the pre-survey, immediately following the EHSA graduation celebration. Pre- and post-intervention survey responses were compared using paired *t*-tests. The University of Missouri Institutional Review Board approved this project (project number 1209741) related to medical student participation and all surveys used. The EHSA intervention for the staff, parents, and children was approved through the UCLA Institutional Review Board (project number 13001703). All medical students and Head Start staff and parents who participated granted written informed consent. This stated agreement to participate in research, that all data would be de-identified, and there would be no negative consequences if they chose to withdrawal from the study.

### Journaling

In addition to the pre- and post-intervention survey, medical students were asked to write journal responses after each EHSA experience. They responded to several open-ended prompts, including a brief description of their encounters with families or staff, how they felt about the interactions, how confident and empathetic they felt during the interactions, what surprised them, and how they might apply what they learned to their future as a physician. Journals were analyzed qualitatively for emergent themes.

### Perspective of Head Start families and staff

Head Start parents and staff were asked to complete a survey about their experience working with the medical students. We believed it was important for a service-learning project of this nature benefit community served as well as students who participated.

## Results and discussion

### Participation

Twenty-two students submitted applications and 12 (*n*=12) were selected to participate in the project (see Table 1). The 12 students had perfect attendance at all required activities.

**Table 1:** Medical student demographics

Total number of students that applied to project	22
Total number of students in project	12
Males	3
Females	9
First year medical students	6
Second year medical students	6
Reported specialty interest • Primary care (pediatrics, internal medicine, family medicine): • Specialty other than primary care:	10 2 (both were Ob-Gyn)
Went to Head Start as a child or had an immediate family member in Head Start	2

### Survey responses

All 12 students completed the pre- and post-surveys. Each question in the survey was designed to evaluate the students’ attitudes, knowledge, or skills confidence regarding health literacy. The characteristics self-reported by each student were compared pre- and post-intervention by a paired *t*-test between average pre-survey to average post-survey (See Table 2).

**Table 2:** Summary of medical student pre- and post- survey response

Questions designed to evaluate	Survey questions	Pre-intervention mean by question/Total mean	Post-intervention mean by question/Total mean	*p*-value of pre/post difference/Total mean
Health literacy attitudes	*On a scale from 1 to 5 (1=not at all important, 5=extremely important) how * ***important*** * is/are:*			
	*1. Health Literacy (HL) to your future career as a physician*	4.75	4.91	0.67
	2. *HL in patient/provider communication*	4.83	4.82	0.67
	3. *Using teach-back as a HL strategy*	4.00	4.85	0.18
	4. *Plain language and clear communication as HL strategies*	4.75	4.82	1.00
	5. *HL training for medical students in their pre-clerkship years*	4.33	4.73	0.17
	Overall attitudes score	4.53	4.68	0.20
Health literacy knowledge	*On a scale from 1 to 5 (1=least, 5=most), how * ***knowledgeable *** *are you about:*			
	*1. Health literacy (HL) issues*	3.33	3.83	0.08
	*2. Role of HL in patient/ provider communication*	3.33	4.5	0.001
	*3. Using teach-back as a HL strategy*	3.33	4.33	0.001
	*4. Using plain language and clear communication as a HL strategy*	3.42	4.42	<0.001
	*5. HL techniques and resources you can use to improve understanding*	3.00	4.17	0.002
	Overall knowledge score	3.29	4.33	0.017
Health literacy skills confidence	*On a scale from 1 to 5 (1=strongly disagree, 5=strongly agree), * ***how do you feel*** * about the following issues:*			
	1. *I am comfortable using teach-back as a HL strategy*	3.25	4.33	0.01
	2. *I receive enough HL training in my current curriculum*	2.67	3.67	0.02
	3. *I feel confident explaining information to Head Start families in a way they can understand*	3.41	4.08	0.02
	4. *I am confident eliciting parents’ questions and concerns about their weight and the weight of their children*	3.42	4.0	0.02
	Overall skills confidence score	3.19	4.11	0.02
Multiple Choice Questions (combined by topic)		Pre-intervention Total % correct	Post-intervention Total % correct	*p*-value of pre/post difference
• *Health literacy definition and identification*		58	58	1.0
• *Childhood nutrition and obesity knowledge*		42	67	0.01
• *Head Start knowledge*		50	67	0.3

Bolded words have increased significance

Students’ perceived attitudes (1=least and 5=most important) about the importance of health literacy were ranked as high both pre- and post-project, with a pre mean=4.53 and post mean=4.68, *p*=0.2. Similarly, there was no change in the students’ ability to define health literacy and identify health literacy issues on multiple-choice questions pre- and post-project. However, students reported statistically significant increases in knowledge (1=least and 5=most knowledgeable; pre mean=3.29 and post mean=4.33, *p*=0.017) and skills confidence (1=strongly disagree and 5=strongly agree; pre mean=3.19 and post mean=4.11, *p*=0.02) related to health literacy techniques, strategies, and resources after participation. Their knowledge about childhood nutrition and obesity issues on multiple-choice questions also improved (*p*=0.01) (See Table 2).

### Journal responses

Journal entries were categorized qualitatively to demonstrate medical students’ insight about their experiences, changed attitudes, growth, and development throughout the project. Two of the authors (BN and CT) used the journaling prompts (i.e., empathy, confidence, surprise) to code applicable segments of the qualitative texts and then used pile sorting to group those codes into themes for qualitative analysis [14]. Additional themes that emerged from the text (i.e., perceptions of families’ health literacy and numeracy, parents’ existing knowledge and motivation) were also coded using open coding based on grounded theory [15]. All of the dominant themes found are presented in Table 3 and exemplar quotations are presented for each theme.

**Table 3:** Summary of qualitative data from student journals

Themes	Sample quotations
Perceptions of families’ health literacy/numeracy	*“I think some of the parents had trouble understanding the nutrition label exercise and I repeated myself in different ways slowly, but you can’t force it. I think most people understood the words I was using, but did not know how to go about finding the answer.”* *“I think some of the math, like understanding the percentages, was hard for the parents. The concept of a portion being part of a whole was difficult.”*
Surprising experiences • Parents’ existing knowledge • Parents’ motivation • Parents’ health-promoting behaviors	*“I…was surprised to see how much they knew.”* *“Many of the parents were much more willing to work on their goals than I had previously expected.”* *“I was pleasantly surprised how many parents were ahead in planning and goal making. All of the parents I met with already had plans for eating healthy and staying active that they had already begun to implement.”*
Personal interactions • Confidence • Empathy • Concerns about families feeling judged	*“I felt confident speaking with families, mainly because they were willing to speak with me and it was more a conversation rather than a lecture or a teaching.”* *“…even though these were low-income individuals, they had the same priorities [for their families] seen in higher-income class individuals.”* *“I’m concerned that the mothers felt I was accusing them of being overweight, rather than sharing the results of the BMI with them.”*

Many students reflected on their first-hand observation of families’ health literacy and numeracy, particularly after students assessed health literacy using the NVS. Students noted the importance of numeracy in addition to literacy when it comes to interpreting nutrition labels, and reflected upon the impact of inadequate health literacy on health-promoting behaviors: “*Their difficulty deciphering the nutrition label made it apparent how hard it would be to eat healthy, if you didn’t know what exactly you were eating. Many individuals struggled with the math, while one struggled with adequately reading the label*.” Related to the theme of health literacy was the idea that the sheer amount of information may have been overwhelming to many of the families: *“*…*so much information was presented to them throughout the training. It was a lot for me to soak in, let alone someone trying to learn it all.*”

Several recurrent themes emerged from the students’ reflections, suggesting most students learned important lessons from their interactions with families. Placing lessons learned into the conceptual model of health behavior change, the students were surprised that some parents had existing health knowledge, were already motivated to make changes, and that many had already started to make healthy lifestyle changes. Of note, several students noted that parents were motivated by their love for their children and their desire to protect their health, which was a surprise for some of the students: “*It made me realize that even if these parents are not necessarily making the best choices for their family with regard to healthy eating and an active lifestyle, it does not mean that they do not love or care for their kids. I think that this is a helpful realization because hopefully this program can use their desire for what is best for their kids as motivation to help educate them and improve their lifestyles in a healthy way*.” Most students realized they could use motivational interviewing techniques to build upon the families’ existing knowledge. Other responses suggest that students may have had previous bias against people who are obese because they believed they were unmotivated, which they found was not necessarily true. Rather than unmotivated, almost all students identified other barriers that prevented the families from attaining a healthy lifestyle.

Finally, many students wrote about their interpersonal experiences with the Head Start families they worked with. The themes that emerged from these reflections were about students’ confidence, empathy, and their concerns about families feeling judged if they were overweight. In general, students reported that their confidence increased considerably with each additional interaction as well as over the course of the project. Several students remarked that they felt increased empathy when they saw how they were similar to the families: *“*…*empathy*…*comes from myself being able to relate to many of the things they are dealing with. I felt like it was very easy to imagine myself in their shoes, because in many ways I am.*” Conversely, one student reflected upon how s/he is different from the families s/he worked with, and how this may affect their interpersonal communications: “*I think the families may have felt uncomfortable talking to me about their nutrition. Here I am a small, fit youngster with a big education trying to convince them to change their lives from how they have been raised.*” That same student went on to say that s/he found the most effective approach was to encourage parents to make small steps and to share his/her own struggles trying to fit in healthy nutrition and physical activity. Several students also commented on how they felt uncomfortable at times discussing BMI with individuals who were overweight or obese, and came to the conclusions that focusing on health, using positive reinforcement and encouragement, and listening to parents’ concerns rather than lecturing, were all effective approaches.

### Head Start’s experience

Surveys about the experience in working with medical students were administered to Head Start parents and staff to evaluate the success of the project from the Head Start community perspective. With 55 surveys completed, all but one ranked their experience with the medical students at a 5 out of 5 in all categories (Table 4). Parents were also asked for additional comments about experiences. Parents responded about their positive experience with the medical students. One parents commented, “It was very rewarding working with the medical student. S/he was very respectful, nice and inquisitive about my family and how s/he could help us. I would recommend everyone get in this program.” Another comment suggested the parents were pleasantly surprised with the experience: “I think I will see doctors a little differently now.” Quantitative surveys revealed that Head Start parents showed statistically significant improvements in their newest vital sign score, nutrition knowledge, shopping behavior, and physical activity. Parent BMIs showed a statistically significant reduction (Table 4). Medical students and the pediatrician faculty advisor provided the Head Start community with a health care provider prospective to enrich the mission of the EHSA program. Addressing health topics with the families and staff in a fun, non-judgmental, interactive way created a non-intimidating atmosphere for parents to ask questions and learn. Additionally, the medical students were able to develop a relationship with the parents and staff over the course of the project, creating a sense of partnership and continuity.

**Table 4:** Head Start parent and staff data

**Survey on Head Start Parent and Staff Experience in Working With Medical Students (** ***N*** **=55)**				
*All questions were ranked on 1–5 Likert scale with 1=none of the time and 5=all of the time*				Post-intervention mean
*1. The medical student made me feel comfortable*				4.98
*2. I felt the medical student understood my questions and comments*				4.98
*3. The medical student talked in terms I could understand*				4.98
*4. The medical student checked to make sure I understood*				4.98
*5. The medical student showed care and concern*				4.98
**Pre/Post Head Start Parent Newest Vital Sign and “Eat Healthy, Stay Active!” Results (** ***N*** **=46)**				
Variable	PRE	POST	Change	95 % CI of Change
Newest Vital Sign Score: mean (SD)	3.6 (2.1)	5.0 (1.2)	+1.4	[1 to 1.9]***
% Adequate Health Literacy	59 %	83 %		
% Possible Low Health Literacy	19 %	17 %		
% High Risk Low Health Literacy	22 %	0		
Body Mass Index: mean (SD) % Obese	32.7 (7.9) 65 %	32.0 (7.7) 61 %	-0.7	[−1 to −0.4]***
Nutrition Knowledge Score	89 %	94 %	+5 %	[0.2-10 %]*
Eating Behavior Score	70 %	74 %	+4 %	[0-7 %]
Shopping Behavior Score	68 %	74 %	+6 %	[2-10 %]**
Physical Activity Score	51 %	63 %	+12 %	[1-23 %]*

*p ≤ 0.05; **p ≤ 0.01; ***p ≤ 0.001

## Conclusion and future direction

This community immersion project was shown to be an effective way for first- and second-year medical students to begin to truly understand the barriers created by poor health literacy and poverty while learning appropriate communication adaptations and health literacy skills crucial to their future success in delivering patient-centered care. Prior to the start of the project, the students’ survey responses reflected attitudes showing the importance of health literacy training for their future careers as health care providers. However, the students reported they did not feel they had adequate knowledge and skills to address these needs. This project offered a unique opportunity for students to not only receive didactic training, but also to learn how to assess health literacy by using a validated tool, such as the NVS, and then put skills learned into practice keeping the patient’s health literacy level in mind. From comparing the pre- and post- survey responses, we demonstrated that student’s knowledge and skills showed statistically significant changes, suggesting this project was able to effectively integrate a voluntary community service project into the curriculum that benefited students. Journal responses were able to capture insightful qualitative changes in attitudes, confidence, and empathy that changed students’ perspective about their future patients. Students gained confidence in their skills as well as better understanding of the needs of their community. Additionally, students and the Head Start community both reported that this was a fun experience. Parent and staff surveys revealed that the Head Start community had a positive experience in working with the medical students. Importantly, the Head Start community benefited from the intervention, shown by significant improvement in parents’ NVS scores and health lifestyle behaviors scores and significant decreased in BMI. This model of health education differs from the traditional didactic classroom format in that it was designed for engagement essential to lead to action. Further, our results suggest the traditional didactics students were already receiving in the classroom had inspired attitudes understanding the importance of health literacy, but had not been able to effectively train future physicians on how to put these attitudes into action. These results suggest a disconnect exists between what is modeled for students in classroom and the very interactive way healthcare professionals are expected to work.

The partnership with Head Start was also beneficial to the community because it enriched the parent education and training and helped to break down barriers between parents and future providers. The project continues to grow; in the second year of EHSA at CMCA, 22 students (10 % of first and second year medical students at MU SOM) are participating in the project. By establishing a partnership between MU SOM and CMCA Head Start, the EHSA program has become a sustainable program within the community.

This study has several limitations. First, it is a very small sample of students who were selected to participate and therefore may not be generalizable to the larger population of medical students (i.e. they were selected through a competitive application and most indicated they were interested in pursuing primary care). Similarly, there was no control group and so we cannot say whether the changes over time were due to participation or to other factors. Limitations in interpreting the results include the possibility that some of the changes in the participants’ knowledge and skills confidence were due to the MU SOM traditional health literacy curriculum. Although the EHSA program resulted in positive changes for Head Start parents, we cannot comment on whether or not medical student participation impacted this because there was no group of parents that did not interact with medical students to serve as a control. In addition, the EHSA program had already been shown to reduce parent BMI from previous studies without medical student involvement [10].

We believe this project is replicable. Medical schools could use this model to develop successful collaborations with local Head Start agencies or different types of community partners. Our study supports conclusions from the study done at Harvard by demonstrating a successful way to teach medical students health literacy skills through building relationships with low-literacy learners to compassionately and effectively provide information [5]. Forming a guiding coalition to help establish a partnership between medical schools and local community agencies was crucial to the success of such a project and gathering local support. The medical student and the pediatrician faculty advisor volunteer support was also essential. The EHSA curriculum was provided by UCLA at no cost. EHSA intervention and medical student project was made possible by a substantial grant and in-kind donations to CMCA Head Start. Due to the success of this innovative partnership in its pilot year, grant opportunities have became available to make EHSA a sustainable part of the Head Start curriculum. The MU SOM and CMCA would welcome the opportunity to share their experience and materials to use as a template for developing a similar curriculum.

Implications for future research include testing intervention components in a larger sample with a control group. Medical students could also be further utilized in the intervention by leading the EHSA parent trainings, providing the students with greater opportunity to present information to an audience using their health literacy skills in addition to meeting with parents one-on-one. The research team plans to re-administer the student surveys at the time of the students’ graduations (for classes 2016 and 2017) in order to see if any differences in exist in the students who participated versus the students who did not.

## Abbreviations

CMCA: Central Missouri community action
EHSA: Eat healthy, stay active!
HCI: Health care institute
HL: health literacy
MU SOM: University Of Missouri School Of Medicine
NVS: the newest vital sign
UCLA: University of California Los Angeles
